# The Pathology of Fever

**Published:** 1899-09

**Authors:** J. Clarence Johnson

**Affiliations:** Atlanta, Ga.; Professor of Physiology and Pathological Anatomy Atlanta College of Physicians and Surgeons


ATLANTA

### Journal-Record of Medicine.

Successor to Atlanta Medical and Surgical Journal, Established 1855,
and Southern Medical Record, Established 1870.

VOL. I. SEPTEMBER, 1899. NO. 6.

BERNARD WOLFF, M.D., EDITOR. M. B. HUTCHINS, M.D., BUSINESS MANAGER.

PUBLISHED MONTHLY. OFFICE 305 AND 306 FITTEN BUILDING.

### *ORIGINAL COMMUNICATIONS.*

### THE PATHOLOGY OF FEVER.

BY J. CLARENCE JOHNSON, M.D.,
Professor of Physiology and Pathological Anatomy Atlanta College of Physicians and Surgeons.

(*Continued from August Number.*)

We have observed that simple irritation of a vaso-motor or inhibitory nerve may, by producing hyperemia of a part, cause increased combustion and generation of heat. We have also seen that the amount of heat in the part diminishes with the subsidence of the active process, and there is no elevation of the body temperature. The question then arises, why is the body temperature elevated when this hyperemia merges into inflammation, and, especially, when there is less oxidation and production of heat in the truly inflamed area.

It may be suggested that it is due to increased combustion in the hyperemic zone. Admitting the plausibility of this view, again it must be asked: Why is not the excess of heat thus generated disposed of as in normal hyperemia of the salivary glands and the liver. The effect, upon the general functions of the nervous system, of the morbid impressions originating within the inflamed area, offers a possible solution to the question, but the insufficiency of the explanation is presupposed by the fact already stated, that the physiological action of a part is accomplished by the associated agency of the blood, nerves and cells.

Bearing in mind that it is cell metamorphosis that gives life and function to every organic substance in the body, we may reasonably look to the protoplasmic compounds for the essential, if not the primary, seat of perversion. So, whatever the cause of inflammation or its accompanying fever may be, its final operation must affect, chiefly, the physical and chemical condition of the cells. The nature and extent of the perversion, and hence the degree and character of fever, will be governed by the kind of tissue involved and the office it usually performs.

The pleura is a serous membrance. Its function is to hold intact and support the lungs, and permit their free and easy movement against the thoracic walls. The efficiency of this function depends upon the elasticity of its connective tissue, and its ability to secrete a fluid for lubrication of its surface.

A person is exposed to a cold draught of air which impresses the sentient filaments of the peripheral respiratory nerves. This impression is conveyed to nerve centers and reflex vaso-constrictor force is discharged to corresponding or adjacent vessels, and the blood is driven to internal collateral branches, and hyperemia of the pleural membrane is the result. If this active determination is not checked, or relieved by nature or art, inflammation follows in quick succession, with all its local and constitutional accompaniments. Whether there was any abnormal condition of the blood, or pleural membrane, that predisposed or rendered it more susceptible than the lungs or any other part of the body to this sudden change of temperature, does not materially concern the point in question now. Whatever the cause and its manner of operation, the essential conditions are the same, and the general rise in the degree of the body heat is like that in any other febrile state that, similarly and to the same extent, impresses the system at large. So it is with any fever of inflammatory origin. The sthenic, asthenic or nervous nature pertains only to the state of body health existing at the time of the attack.

In attempting, therefore, to define the pathology of inflammatory fever, we are thus far safe only in saying that, primarily occurring, it is the result of perverted vascular action through vitiation of the blood, morbid impressions upon the nerves, or injury to the part, which modifies the osmotic interchange and inhibits or disorders the metabolic functions of the cells, leaving in doubt the manner in which these local changes produce an elevation of the general temperature.

Even were this explanation sufficient, it would not comprehend the phenomenal rise of temperature usually classed as secondary fever.

Referring to pleuritic inflammation, should the serous fluid exuded fail to be absorbed with resolution or decline of the inflammatory process, putrefaction or decompostion occurs, and this change is announced by a chill, and sudden increase in body heat. What has produced this complication? It is obviously due to the chemical action and change of state of the fluid serum, or to impressions made by it upon the nervous system—aided, perhaps, by the materies morbi which influenced or followed the transformation into pus—else there is a loss or addition of latent heat; for the process is independent of physiological action, as there is no increased tissue metamorphosis, beyond that which led to its effusion.

But suppuration is not always attended by rise of temperature, and we must extend our enquiry beyond the local action or conduct of a given cause.

If all fevers were of a secondary nature, that is, dependent upon a primary local lesion, it would further encourage the idea that it is the behavior of the nervous system that causes the fever. But there are fevers in which the local change is secondary to the constitutional disorder, and plays but little part in its production, being, alike with the fever, dependent upon a common cause.

While the history of these local changes constitute a part of the pathology of the fever and throw important light upon the path of our investigation, they are so near akin to those of ordinary inflammation, induced by a perversion of the same functions and involving essentially the same factors, the field of research must necessarily broaden as the operation of the exciting cause becomes more extensive.

The only appreciable difference between ulceration of Peyer's patches in typhoid fever or hyperplasia of other intestinal lymphoid tissue, and that induced by simple enteritis, is in the extent, severity and duration of the process. So the very similarity of local action in primary and secondary fevers, and the relation which this action in both sustains to the general functions, veils with deeper obscurity the nature of the effect which the impressions they create must have to produce a similar condition of heat and temperature in the body.

The fact that essential fevers, as typhoid, scarlet fever and diphtheria, are produced by specific micro-organisms, would, naturally, lead to the study of the several characteristics of these germs, with the view of better understanding their conduct within the body, but it is not within the province of this paper to dwell upon them further than is necessary to determine their relation to organic existence, and the chemical changes which they favor or induce. Revelations of the culture tubes have been invaluable aids to diagnosis, prognosis, prophylaxis and therapeutics, and have given us clearer insight into the distinctive features of these parasites, but the knowledge thus acquired as yet, cannot comprehend or satisfactorily explain their behavior within, and effect upon, the human organism.

For our present study, it is sufficient to bear in mind that they are of vegetable origin, having an independent life, as all other organic units, and, such being true, must possess a selective, catalytic and eliminative function.

The resistance which they have to extremes of heat and cold demonstrates the fact that, though allied, they are essentially different in their chemical nature from the vegetable proximate principles in the body, and their catalytic power likewise shows their kinship to the proteids or plasmic bases, and, hence, they must have different organic life. Some are not destroyed by freezing, while others can live in a temperature barely short of 212° F., while 160° F., in the human body, would coagulate the proteids, and 83° F. is the lowest it can tolerate.

While this marked discrepancy exists between the susceptibility of bacteria and the human body to thermic changes, it is, nevertheless, true that the bacteria flourish most at or near the normal temperature of the body.

The manner in which these germs enter and leave the body aids us somewhat in studying their influence and mode of action. Those of typhoid fever, for example, are introduced by the ingesta, water and milk being their favorite vehicles, and, if they successfully resist the digestive changes and establish a residence in the system, the invasion is followed by a rise of temperature. But not every one into whose body they are introduced succumbs to their vitiating influence. Nor does every one who overcomes them once enjoy final exemption from them. It is a fact of common observation that with this, as with other infections or contagious fevers, an individual may repeatedly escape a known contagion and subsequently yield to it.

So there must, at the time of the attack, exist, in the system, some disturbance of the vital energy which predisposes to the morbid action of the germs and the dissemination of their products. This may be vaguely defined as a lowered state of nutritive and functional activity, or a peculiar habit or condition of the body which renders its powers of physiological resistance less. Again, some of these diseases attack at one period of life, while other periods are immune.

A complete and full comprehension then, of the pathology of essential fever involves a knowledge of the nature of the inherent condition, or the perversion which predisposes to their development. The fact that some days often elapse before the presence of the disease is manifest, or the temperature rises, presupposes the fact that the germs themselves are multiplied within the body or that their effect is accumulative.

Typhoid fever has an incubation of twelve to twenty-one days. That they impress the system from the time of their entrance is proved by the disturbances of digestion, secretion and animal function during incubation. Eventually, with or without a chill, there is a sudden or gradual rise of temperature—the sufferer has typhoid fever. With the insidious development of the disease there has been a gradual diminution of material usually supplied the body for heat production, and yet, with the onset, the degree of heat is higher—the amount depends upon the extent to which the specific capacity of the proximate principles, pre-existing in the body, have been exhausted.

It is worthy of observation, that while these bacteria differ in their physical natures and their affinity or preference for certain parts of the body as seats for local action, and, while the nature of this action also varies, the general nutritive disturbance corresponds very nearly in them all. Thus, in typhoid fever, diphtheria and scarlet fever, there exists in all the cells of the body a state known as cloudy swelling, resembling somewhat fatty infiltration, or fat necrosis. Whatever the exact histological or chemical changes that induce this may be, it is an evidence of perverted or inhibited metabolism, attended by great functional debility. That this cloudy swelling is a protoplasmic disorder, there is no doubt, and that it is akin to fatty infiltration, or degeneration, is evidenced by the fact that not only does it present similar microscopical appearances, but it is induced and attended by the immediate pathological condition and processes that cause these forms of degeneration.

Notwithstanding the increased evolution of heat, the material of the plasmic substance itself, or the pabulum which it is wont to transform, fails to be perfectly oxidized, as is the case in fatty degeneration. However, we must not forget the fact that regardless of this swelling of the cell capsule and its substance, and the change in its chemical or histological arrangement and physiological action, its potential energy or amount of specific heat may have, and probably has, increased. Then, if the oxidizing process continues sufficient to produce the excessive amount of heat which causes the fever, it would appear that the process takes place outside the cells and independently of their chemico-vital agency.

It has already been stated that there is but little combustion in the blood, but, for the present, granting that, in the absence of cellular oxidation, the process does take place within the blood, it devolves upon us to determine how these bacteria have retarded or modified the metamorphosis of cells; and why, if they do, the same influence is not exerted in the blood. The theory has been advanced that they prevent the union of oxygen with the fats and carbohydrates, or prevent the cells from selecting and appropriating this class of proximate principles. If this is true, it further removes the probability that it is the chemico-vital action of the cells that is responsible for increased heat formation, inasmuch as these principles are chiefly employed as fuel for combustion, and they exist in small proportion in the cells.

It still may be urged that the exclusion of these principles does not weaken the argument that combustion takes place within the cell, as in normal tissue metamorphosis, since the same process occurs in the muscle protoplasm during contraction. Yet it is a matter of dispute, as before suggested, whether it is the muscle protoplasm that is oxidized in contraction. The presence of sarcolactic acid within the cells, after exercise, favors the view, that the protoplasm simply employs the force supplied by combustion, chiefly of carbohydrates and fats, and that the increased heat incident to the exercise is due to this chemical change. This seems a more reasonable explanation of muscular action, for it is not logical or consistent to believe that the liver cells would be furnished material for the performance of their function and the muscle be denied the same. Besides, in the treatment of fevers, the starchy and oily foods are usually withheld, so the amount contained within the cells at the incipiency of the attack would be exhausted long before the fever had run its course.

The correctness or incorrectness of this theory, then, opposes no obstacle to the determination how bacteria inhibit or pervert the catalytic or nutritive function of the cells. There are some adherents to the idea that these infectious diseases are due to a specific poison, and that the germs usually found in the products of decomposition are a consequence and not a cause. Whatever the *materies morbi* of these diseases may be, that it does not act as a mere poison is evidenced by comparison of its effect with the toxic effect of drugs that simply depress or excite the vital energy, with little or no appreciable organic change.

It is true, there are drugs which produce similar histological or pathological conditions in the protoplasm of the cells, as arsenic or phosphorus. Yet, in the former the temperature is elevated, and in the latter it is lowered—an inconsistency, which proves the essential and distinctive nature and action of each exciting agent in the class of diseases named.

But it is insisted, not every one into whose system these germs are introduced, or in which they are found, has or develops the disease which they are supposed to cause; we have already made mention of this fact. It is true that staphylococci are found in healthy as well as diseased bronchial mucous membranes, and other anomalous facts might be stated in connection with their life and conduct in the body, but it has been satisfactorily demonstrated by inoculation of these germs that they produce a disease similar in local and constitutional character whenever they attack susceptible tissue, and that they are nearly always present in the blood or tissues, or in both, in the diseases to whose pathology they are supposed to bear a causative relation. So we can well afford to leave the opponents of the germ theory of fever to their doubts and speculations and accept facts which have been established by careful physiological and pathological experimentation.

Therefore, in seeking to determine the causative relation of the Klebs-Lœffler bacillus to diphtheria, or the pneumococcus to pneumonia, we may study their action as organized agents affecting the nutritive and functional activity of the tissue they involve, after the manner that we would study the action of ptyalin on starch, pepsin on proteids, or the catalytic agent within the cell; and the difference in their action from the action of those ferments will afford at least a clue to the character of the perversion which leads to the local and general pathological changes, viz.: the pseudo-deposit in the throat, and toxalbumin in the blood, in the one, and the inflammation in the lungs in the other, with the abnormal rise of temperature in both.

Inasmuch as any agent, acting in any manner upon the body must employ the *principium vitæ* of the body, either by inhibition, excitation or perverson, and as the vital force is the result of dissociation of proximate principles, and is the equivalent of potential energy or heat which this dissociation evolves, however these morbid agents act, they must disturb the relation existing normally between the kinetic force and heat, or interfere with the conversion of one into the other.

Aside from the theories suggested with reference to their mode of action, let us look for a moment at their known effect. Serum-therapy is based upon the supposition that the germ of the disease to which it is applied so invests the plasma of the blood with its toxic properties that, if injected into another organism, it will have an immunizing or antitoxic effect, as the lymph of variola, either by producing a condition somewhat similar to the original disease or by lessening the natural predisposition to the operation of the cause. This is accomplished by the serum exhausting fully, or in a measure, the innate susceptibility, or augmenting the power of vital resistance.

It is a physiological fact that the intestinal bacteria are capable of, and do cause, a more complete transformation of the proteids than pepsin or trypsin—skatol, phenol and indol being their usual products. It is also a fact that these products are antiseptic, and destroy the bacteria whose fermentative action gives them existence. The same chemical change, doubtless, occurs in a measure within the proteids of the blood and cells. Hence the opinion prevails that it is the toxines, or the unformed ferments, the product of these germs, that cause the abnormal temperature.

This theory, however, is not tenable. The fact that serum-globulin and serum-albumin are so changed presupposes the fact that they are unfitted for functional and nutritive purposes. In consequence the activity of the cells must suffer, and their elective and catalytic power is impaired. We have noted the fact that normal secretion and excretion depend upon a normal osmotic action, and that in this action the nerves, blood and cells are all engaged, and that one cannot act perfectly without the others.

Therefore, this change in the plasma of the blood opposes the osmosis, and there is no chemical, physical or physiological process which can otherwise accomplish it. A carbohydrate is changed into glucose by the assumption of one molecule of water under the catalytic influence of ptyalin or amylopsin. Oxidation of this sugar within the system produces heat, water and $CO_2$. This is nature's law, and nature's laws are invariable.

As with sugar so it is with the proteids, so far as chemico-vital changes are concerned. By their union with oxygen kreatin, kreatinin, urea or uric acid, with heat and $CO_2$, are formed, and this transformation is accomplished by the anabolic and catabolic power of the cells, and the aggregate of this power is the measure of kinetic or vital capacity of the body.

It is evident that if the theory of combustion as the source of heat is correct, we are confronted by several difficulties in explaining the increased production as a cause of essential fevers. As before stated, the amount of combustion depends not only upon oxidizable material but upon the amount of oxygen supplied.

It is maintained by pathologists that these bacteria hinder the red-blood corpuscles from giving up their oxygen to the tissues, and it is also stated that the leucocytes, or progenitors of red-blood disks, have a special affinity for these bacteria and destroy them or arrest their action. It is also known that bacteria reduce the size and number of red-blood corpuscles in the blood.

Leaving for a moment the consideration of the bacteria in the relation to the blood, let us ascertain, if possible, how they influence cell action directly. That some do penetrate the cell capsule and enter the cell substance there is no doubt, and that they there produce a change in the protoplasm is equally probable; but the question most pertinent is, how do they produce this change? If the foregoing deductions are correct, the cloudy swelling, atrophy and degeneration of the cells would naturally follow independently of the direct action of the germs, as this condition is inevitably a consequence of deficient pabulum or deficient oxidation. This is doubtless the true explanation of the morbid cellular state present in diphtheria and kindred diseases. When the germs enter the cells, it is easily apparent how their fermentative action would effect the same chemical changes in the constituent element that they do in the blood, the cells and blood having, so far as their elementary principles are concerned, the same composition. In the light of these facts, and the laws of physiological action, increased heat production after the usual habit of the body seems impossible.

Yet, without increased tissue metamorphosis, we are left with the difficulty of explaining the source of the increased amount of $CO_2$ and urea which is eliminated in fever.

A reasonable, if not an entirely satisfactory, explanation, however, is afforded by facts before mentioned. We have seen that the liver generates a great amount of heat, urea and $CO_2$. $CO_2$ is also formed by decomposition of the carbonates in the blood and tissues. Urea is the imperfectly oxidized product of proteids, and hence an increase above the normal 450 to 500 grains daily indicates increased metamorphosis of this class of proximate principles.

But Voit has shown, by experiment upon fasting animals, that an increase of urea, together with the cloudy swelling and fatty degeneration which mark the pathological anatomy of essential fever, may be induced readily by the administration of phosphorus; notwithstanding the decrease in the oxidizing process incident to absence of food and reduction of oxygen supplied. This presupposes the fact that cell albumin is capable of splitting up into urea, fat and oily products, independent of natural physiological action. Hence we may reasonably conclude that the increase in $CO_2$ and urea is due more to deficient rather than to excessive combustion.

It is true that there is a gradual loss of weight and substance of the body accompanying these changes, and the loss is due to the natural excretory functions. Yet, it is at the same time true that less food is being taken into the body, and the natural combustive process is conserved or reduced as far as the maintenance of economical physiological action permits.

Besides, considering again, for a moment, the febrile state, we find that every factor supposed to be most actively engaged in producing heat, water and $CO_2$, is comparatively quiescent. The only muscles actively engaged are those of respiration and circulation, and the glandular system is also in a state of partial rest, as is evidenced by diminished secretion and excretion. The blood is also less capable of supplying oxygen for combustion.

It has been demonstrated that an average man requires 2½ pounds of food per diem, or the equivalent of 4,630 grains of carbon and 300 grains of nitrogen, and that the heat generated within 24 hours is 7286.4 units, or 3,312,000 small calories. Hence it is apparent that if waste and repair are equal, 4,630 grains of carbon and 300 grains of nitrogen must be oxidized daily, and inasmuch as the union of oxygen with other elements is always constant and the heat product the same, whether by slow or rapid combustion, if increased oxygen is the source of fever, the product eliminated, $CO_2$, $H_2O$ and urea ought to correspond with the heat units necessary to raise the temperature to the degree it registers. This relation is not maintained.

It is evident that while fever or abnormal elevation of the temperature is the result of a chemical process, and that this chemical process consists in the transformation of energy into heat, or the liberation of latent heat or potential force, that does not presuppose the fact that it is altogether the result of oxidation after the usual manner of tissue combination. If that were true, and true by virtue only of the performance of the vital functions, and this was the only factor, then it would naturally follow that every part of the body—the cutaneous, as well as the internal—would participate in the performance of these functions which gave them alike a greater amount of heat and a higher temperature. Such being true, heat elimination would be commensurate with heat production, as regards the excess generated.

So, we must look beyond the changes in the blood and in the cells, and the process of combustion, as the sole factors in the pathology of fever.

*(To be continued.)*

